# Associations between Short-Term Air Pollution Exposure and the Peripheral Leukocyte Distribution in the Adult Male Population in Beijing, China

**DOI:** 10.3390/ijerph20064695

**Published:** 2023-03-07

**Authors:** Yuting Xue, Ji Cong, Yi Bai, Pai Zheng, Guiping Hu, Yulin Kang, Yonghua Wu, Liyan Cui, Guang Jia, Tiancheng Wang

**Affiliations:** 1 Department of Laboratory Medicine, Peking University Third Hospital, Beijing 100191, China; 2 Department of Epidemiology, School of Public Health, Peking University, Beijing 100191, China; 3 Department of Occupational and Environmental Health Sciences, School of Public Health, Peking University, Beijing 100191, China; 4 School of Medical Science and Engineering, Beihang University, Beijing 100191, China; 5 Beijing Advanced Innovation Center for Big Data-Based Precision Medicine, Beihang University, Beijing 100191, China; 6 Institute of Environmental Information, Chinese Research Academy of Environmental Sciences, Beijing 100012, China

**Keywords:** air pollution, short-term exposure, peripheral leukocyte, adult, male, China

## Abstract

The inflammatory effects of air pollution exposure may account for increased public health risk. However, evidence regarding the effects of air pollution on peripheral blood leukocytes in the population is inconsistent. We investigated the association between the short-term effects of ambient air pollution and the peripheral blood leukocyte distribution in adult men in Beijing, China. From January 2015 to December 2019, a total of 11,035 men aged 22–45 years in Beijing were included in the study. Their peripheral blood routine parameters were measured. The ambient pollution monitoring parameters (particulate matter ≤ 10 µm (PM_10_), PM_2.5_, nitrogen dioxide (NO_2_), sulfur dioxide (SO_2_), carbon monoxide (CO), and ozone (O_3_)) were collected daily. The potential association between ambient air pollution exposure and peripheral blood leukocyte count and classification was analyzed with generalized additive models (GAMs). After adjusting for confounding factors, PM_2.5_, PM_10_, SO_2_, NO_2_, O_3_, and CO were significantly correlated with changes to at least one peripheral leukocyte subtype. Short-term and cumulative air pollutant exposure dramatically increased the participants’ peripheral blood neutrophil, lymphocyte, and monocyte numbers and decreased eosinophils and basophils. Our results demonstrated that air pollution induced inflammation in the participants. The peripheral leukocyte count and classification can be utilized to evaluate the inflammation induced by air pollution in the exposed male population.

## 1. Introduction

Following rapid economic development and the continuous advance of urbanization, air pollution has become a serious challenge in the history of human progress [1]. Research evidence demonstrated that air pollution exposure is one of the most crucial risk factors in global public health and has attracted increasing attention from public health researchers [2]. Worldwide, long-term air pollution exposure is responsible for 4.2 million–8.9 million deaths [3,4], and the number is increasing. The quantity of research has demonstrated that air pollution exposure is closely related to diverse diseases in multiple systems, which seriously affect quality of life and happiness [5]. Continuous air pollution exposure elevates the risk of cardiovascular disease [6], chronic obstructive pulmonary disease [7], chronic kidney disease [8], intestinal disease [9], and central nervous system disease [10]. Furthermore, research evidence has also suggested that air pollution exposure may increase male infertility risk [11,12], possibly by affecting sperm development, which in turn exerts deleterious effects on sperm count and quality [13].

Exposure to air pollution, especially particulate matter (PM), induces systemic inflammatory responses [14,15,16,17,18] that lead to the release and redistribution of peripheral white blood cells (WBCs) [19], which may be a critical factor in the effect of air pollution on human health. Nevertheless, earlier studies on the association between air pollution and peripheral WBC count were inconsistent, which might have been due to study population differences or relatively small samples and the lack of the assessment in consideration of the potential effects of confounding factors.

The objective of the present study is to investigate the association between short-term air pollution exposure and the peripheral blood WBC count and classification in adult males and explore the potential influence of air-pollution-induced inflammation.

## 2. Methods

### 2.1. Study Population

The study population comprised people who visited the Peking University Third Hospital Medical Center from January 2015 to December 2019. The participants’ data were screened according to the established entry criteria. The study population was limited to those aged 22–45 years. Patients with systemic diseases such as liver, renal, cardiovascular, hematological, or endocrine disorders or any infections or inflammation and patients with abnormal clinical laboratory indicators (biochemistry, immunology, endocrine hormones, or hematology) were excluded from the study to eliminate the interference of confounding factors, such as disease, as much as possible. Finally, 11,035 men were enrolled in the study. This study received approval from the Peking University Third Hospital Medical Science Research Ethical Committee.

### 2.2. Blood Collection, Processing, and Measurement of Peripheral Blood Leukocyte Count and Classification

The peripheral blood samples were obtained by venipuncture and collected in vacuum blood collection tubes containing EDTA-K_2_ as the anticoagulant (INSEPACK^®^, Sekisui, Beijing, China). The peripheral leukocytes were counted and classified into neutrophils, eosinophils, basophils, lymphocytes, and monocytes in the traditional five-subtype classification method using an automatic blood count analyzer (SYSMEX XN-2000 Automated Hematology Analyzer, Kobe, Japan).

### 2.3. Environmental Variables and Meteorological Data

The Beijing air pollution data from January 2015 to December 2019 were collected from the China Meteorological Administration and the Environmental Monitoring Center (http://www.cnemc.cn/, accessed on 1 March 2020), which records hourly measurements of particulate matter ≤ 2.5 µm and ≤10 µm (PM_2.5_ and PM_10_, respectively), sulfur dioxide (SO_2_), nitrogen dioxide (NO_2_), ozone (O_3_), and carbon monoxide (CO). The air pollutant concentrations were converted into 24 h averages for further analysis. The climatic data (daily averages of temperature, relative humidity, wind speed, and air pressure) during the same period were collected from the China Meteorological Data Service Centre (http://data.cma.cn/, accessed on 1 March 2020). The daily average value of each meteorological indicator was calculated by hourly observational data.

### 2.4. Statistical Analysis

Descriptive statistics were conducted for the peripheral leukocyte count and classification and ambient environmental measurements. Data normality was tested with the one-sample Kolmogorov–Smirnov method. Spearman correlation analysis was performed for bivariate correlation analyses between meteorological factors and air pollutant parameters. The effect of air pollution on the peripheral leukocyte count and classification was estimated with the generalized additive model (GAM). The GAM is a linear predictor of semi-parametric models that contain a series of covariates with additional nonparametric smooth splines. The GAM demonstrates greater flexibility and fewer assumptions compared with ordinary least squares (OLS) linear regression. Furthermore, the GAM remains valid even if the data do not satisfy OLS assumptions such as normality and uniformity. Additionally, the advantage of the GAM is its ability and flexibility to address different nonlinear effects [20]. In the analysis of the effect of short-term air pollution exposure on peripheral blood leukocyte distribution, it was necessary to control the influence of meteorological factors in the time series, which included the ambient temperature, humidity, air pressure, wind, and confounding factors such as season (heating and non-heating) and the study population age. Moreover, the effects of the single-day and average lag effects of air pollutants on the dependent variable were considered. Therefore, we fit the models with different lag periods from lag of 0 days (the day of the peripheral blood leukocyte analysis) to lag of 3 days. To consider the exposure effects of the average over the same and previous days, the association between air pollution and peripheral leukocyte parameters was estimated with the 1–3-day moving averages of air pollution parameters. The covariates were adjusted and normalized with log_10_ transformation. All parameters were calculated as percent changes with 95% confidence intervals (CIs) associated with the change in SO_2_, NO_2_, PM_2.5_, PM_10_, O_3_, and CO daily average concentrations.

The specific model is as follows:

$$\log\left[E\left(Y_{i}\right)\right]=\beta_{0}+\beta X_{i}+s_{\left(\mathrm{Age},df\right)}+s_{\left(T,df\right)}+s_{\left(H,df\right)}+s_{\left(W,df\right)}+s_{\left(P,df\right)}+as.factor\left(DOW\right)+\varepsilon$$

where $E\left(Y_{i}\right)$ represents the expected peripheral blood leukocyte count and its classification on the observation data of the *i*th observation sample; $\beta_{0}$ is the intercept of this model equation; β is the regression coefficient, where the practical significance of its value is that every 1-unit increase in the independent variable can alter the dependent variable by 100*β*%. $X_{i}$ represents the average daily air pollutant concentration; $s_{\left(\mathrm{Age},df\right)}$ refers to the age spline smoothing function; $s_{\left(T,df\right)}$ refers to the temperature spline smoothing function; $s_{\left(H,df\right)}$ refers to the humidity spline smoothing function; $s_{\left(W,df\right)}$ refers to the wind speed spline smoothing function; $s_{\left(P,df\right)}$ refers to the barometric spline smoothing function; as.factor(DOW) is a set of dummy variables on seasonality added to adjust the influence of the heating and non-heating seasons in Beijing; and $\varepsilon \sim \left(0,\sigma^{2}\right)$ refers to the residual. The effect of short-term air pollution exposure on the participants’ WBC distribution was observed through single-factor GAM analysis.

Two-sided P-values were reported and considered statistically significant when <0.05. The Statistical Package for the Social Sciences (SPSS) 25 (SPSS Inc., Chicago, IL, USA) was used for general statistical analysis. The GAM was performed with the mgcv package, and the data were visualized with the packages corrplot (version 0.84) and ggplot2 (version 3.1.1) of R (version 4.3.0, R Project for Statistical Computing) and RStudio (version 1.2.1335), respectively.

## 3. Results

### 3.1. Descriptive Statistical Analysis of Beijing Meteorological Factors and Air Pollutants and the Peripheral Blood Leukocyte Distribution of the Study Population

Figure S1 depicts the time series analysis of the average daily concentration of air pollutants in Beijing from January 2015 to December 2019, which included the observed average daily concentrations of PM_2.5_, PM_10_, SO_2_, CO, NO_2_, and O_3_ and the time trend, seasonal fluctuation, and random error. Except O_3_, the other pollutants demonstrated an obvious decreasing trend since early 2017, which was consistent with the effects of stricter environmental protection policies in Beijing in 2017. Furthermore, all air pollutants demonstrated obvious seasonal fluctuations, namely at the beginning and end of each year. In addition to the relatively higher daily average O_3_ level, these times of year are the Beijing municipal heating period of each year, and therefore, the above results suggested that air pollutant cyclical fluctuations might be correlated with the heating period in Beijing. Consequently, it was necessary to add a seasonal dummy variable to the GAM analysis and divide the whole year into two heating and non-heating periods to adjust for the influence of seasonal factors on the participants’ peripheral blood leukocyte distribution.

Regarding the peripheral blood leukocytes, the logarithm values for the participants’ counts of the five WBC categories demonstrated that they approximately obeyed normal distribution by fitting its frequency distribution diagram.

Table 1 depicts the descriptive statistical results of the participants’ WBC distribution and the Beijing meteorological factors and air pollutant parameters from 2015 to 2019. The mean participant age was 30.38 years, and the participants’ average peripheral blood total WBC, neutrophil, eosinophil, basophil, lymphocyte, and monocyte counts were 6.77 × 10^9^/L, 3.94 × 10^9^/L, 0.03 × 10^9^/L, 0.13 × 10^9^/L, 2.21 × 10^9^/L, and 0.36 × 10^9^/L, respectively. The mean daily temperature, humidity, wind speed, and air pressure in Beijing from 2015 to 2019 were 13.95 °C, 49.88%, 2.032 m/s, and 1012 Pa, respectively. From 2015 to 2019, the average daily PM_2.5_, PM_10_, SO_2_, CO, NO_2_, and O_3_ concentrations were 59.9 μg/m^3^, 84.42 μg/m^3^, 8.064 μg/m^3^, 0.99 mg/m^3^, 43.68 μg/m^3^, and 96.86 μg/m^3^, respectively.

### 3.2. Spearman Correlation Analysis between the Meteorological Index and Air Pollutants

The Spearman correlation analysis demonstrated that all air pollutants except O_3_ were positively correlated, while O_3_ was significant negatively correlated with the other air pollutants (*p* ≤ 0.05). CO and PM_2.5_ were the most strongly correlated (correlation coefficient = 0.86), followed by PM_2.5_ and PM_10_ (correlation coefficient = 0.80). Among the meteorological factors, temperature and wind speed were negatively correlated with all air pollutants other than O_3_, and most of them had statistical significance (*p* ≤ 0.05). The bivariate correlation analysis suggested serious multicollinearity among the meteorological indicators and air pollutant parameters (Figure S2).

### 3.3. Generalized Additive Analysis of the Single-Factor Pollution Model

Based on the earlier results, it was appropriate to select the connection function based on the normal distribution in the GAM. After adjusting for the participants’ age and the confounding effects of temperature, humidity, wind speed, and atmospheric pressure, the generalized additive analysis of the single-factor air pollution model demonstrated that PM_2.5_, PM_10_, SO_2_, CO, NO_2_, and O_3_ were strongly correlated with at least one leukocyte subtype. We constructed a forest map based on the results of the generalized additive analysis of the single-factor pollution model, in which the point and CI line were converted from the estimator *β*. The point value was $100\times \left(e^{\beta }-1\right)$, and the upper and lower CI limits were $\left(e^{\beta \pm 1.96SD}-1\right)$, which indicated that the air pollutant concentration in each additional unit could cause a change in the leukocyte count percentage and the corresponding 95% CI. Consequently, the absence of intersection between the 95% CI line and the straight line of 0 indicated that the association between air pollutants and the peripheral blood leukocyte classification count was statistically significant at a confidence level of 0.05.

**Figure 1:**
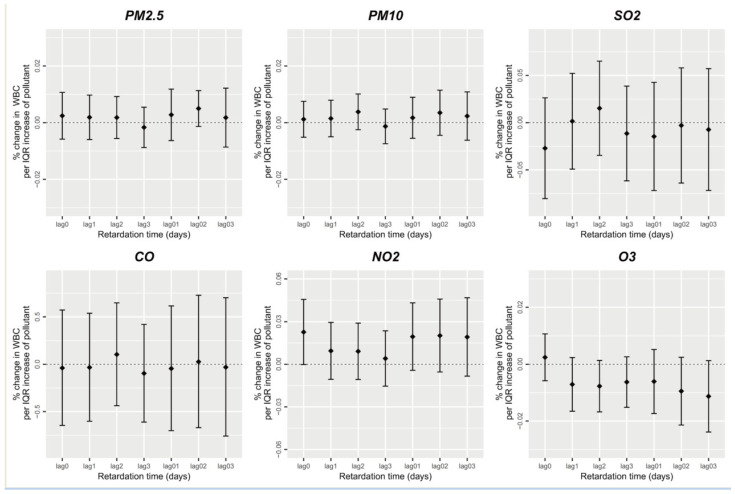
Short-term exposure to air pollutants and leukocyte count.

The association between short-term air pollutant exposure and leukocyte count was assessed with a single-factor pollution model. Figure 1 demonstrates that in short-term air pollution exposure, in the 0–3-day lag, PM_2.5_, PM_10_, SO_2_, CO, NO_2_, and O_3_ exerted non-significant single-day lag effects and average lag effects on the WBC count, which indicated that there was no significant correlation between air pollution exposure of 0–3 day lag and the participants’ total peripheral blood WBC count (*p* > 0.05).

**Figure 2:**
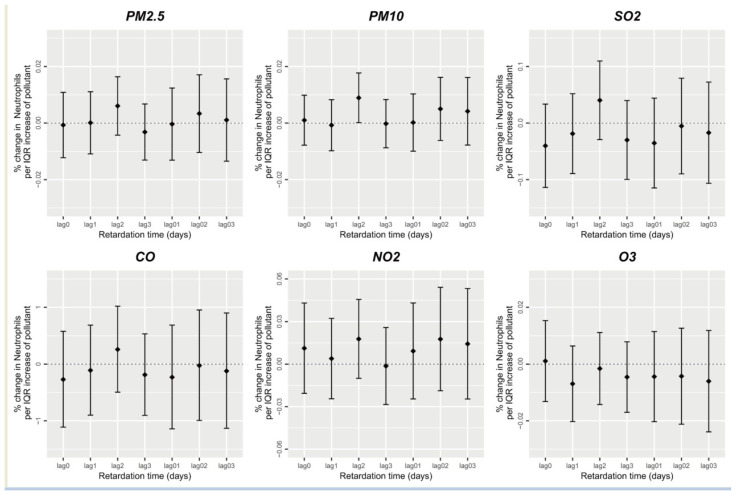
Short-term exposure to air pollutants and neutrophil count.

Figure 2 depicts the association between short-term air pollutant exposure and neutrophil counts. The effect was similar to that on the total WBC number, except that PM_10_, PM_2.5_, SO_2_, CO, NO_2_, and O_3_ exerted no significant single-day and average lag effects on the participants’ 0–3-day-lag peripheral blood neutrophil counts. PM_10_ was significantly positively correlated with the peripheral blood neutrophil numbers only on 2-day lag (estimated β = 8.916 × 10^−5^). It was suggested that each unit increase in 2-day-lag PM_10_ concentration increased the expected participants’ peripheral blood neutrophil count value by 0.0089% (95% CI: 0.0001–0.0177%).

**Figure 3:**
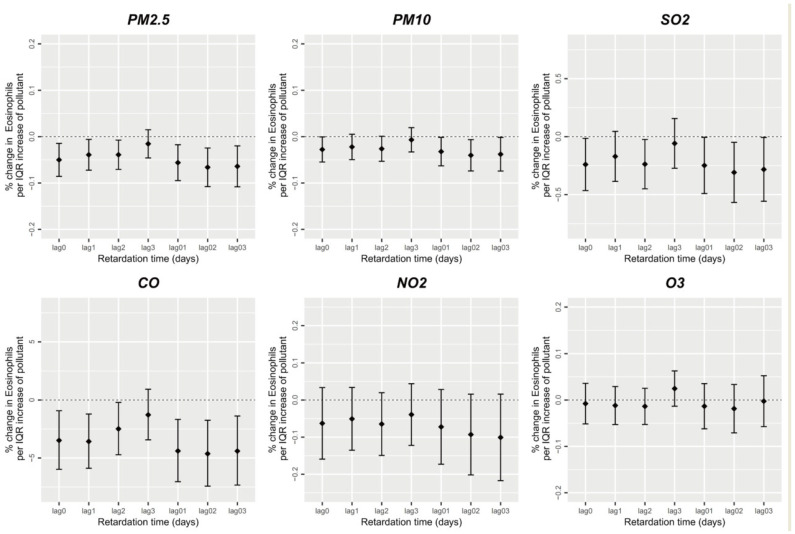
Short-term exposure to air pollutants and eosinophil count.

There was a significant association between short-term air pollution exposure and peripheral blood eosinophil counts. Notably, short-term PM_2.5_, PM_10_, SO_2_, and CO exposure was significantly negatively correlated with peripheral blood eosinophil counts (*p* < 0.05) (Figure 3), while short-term NO_2_ and O_3_ exposure was not significantly correlated with peripheral blood eosinophil counts (*p* > 0.05). The single-day lag effect of short-term PM_2.5_, PM_10_, SO_2_, and CO exposure on peripheral blood eosinophils was strongest on 0-day lag, then decreased slowly until 3-day lag, while the mean lag effect on peripheral blood eosinophils peaked at lags of 0–2 days. Our results demonstrated that when PM_2.5_, PM_10_, SO_2_, and CO exposure increased by one unit on the same day, the participants’ peripheral blood eosinophils decreased by 0.050%, 0.028%, 0.24%, and 3.485% (*p* < 0.05), respectively. Simultaneously, the average PM_2.5_, PM_10_, SO_2_, and CO exposure increase by one unit in 0–2-day exposure was accompanied by 0.066%, 0.040%, 0.309%, and 4.633% decreased peripheral blood eosinophils, respectively (*p* < 0.05).

**Figure 4:**
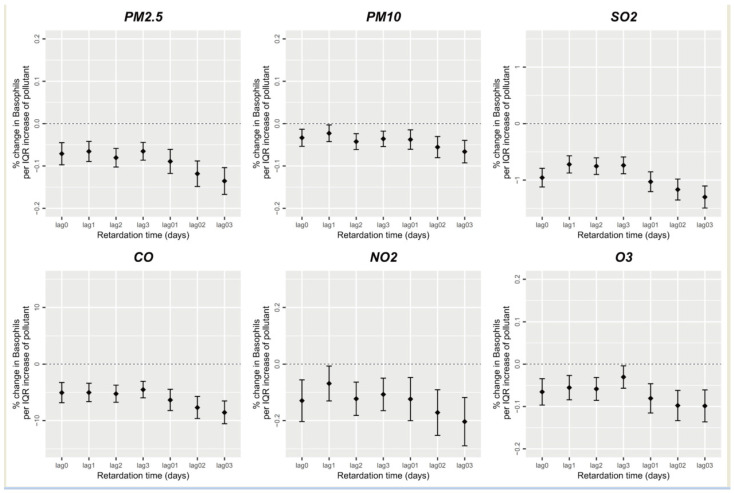
Short-term exposure to air pollutants and basophil count.

Figure 4 depicts the association between short-term air pollution exposure and peripheral blood basophil counts. Short-term exposure to PM_2.5_, PM_10_, SO_2_, CO, NO_2_, O_3_ and other air pollutants was significantly negatively correlated with peripheral blood basophil counts (*p* < 0.05). The average lag effect of short-term air pollutant exposure was generally greater than the single-day lag effect. Furthermore, the average lag effect of short-term air pollutant exposure was greatest with lags of 0–3 days. An increase in one unit in the average PM_2.5_, PM_10_, SO_2_, CO, NO_2_, and O_3_ concentrations of lags of 0–3 days caused the peripheral blood basophil counts to decrease by 0.136%, 0.066%, 1.300%, 8.559%, 0.204%, and 0.099%, respectively (*p* < 0.05).

**Figure 5:**
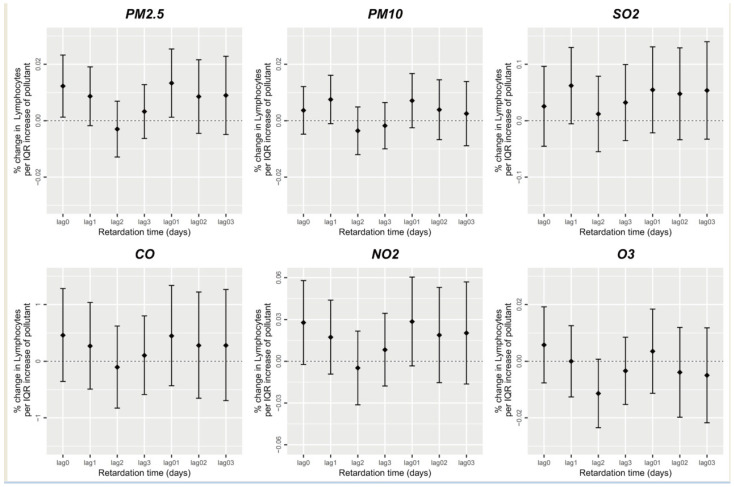
Short-term exposure to air pollutants and lymphocyte count.

Figure 5 illustrates the association between the participants’ absolute peripheral blood lymphocyte values and short-term air pollution exposure parameters. Only PM_2.5_ exerted a significant effect on peripheral blood lymphocytes, where each unit increase in the PM_2.5_ average concentration resulted in a 0.012% increase in peripheral blood lymphocytes (*p* < 0.05), while short-term PM_10_, SO_2_, CO, NO_2_, and O_3_ exposure had no significant effect on peripheral blood lymphocytes (*p* > 0.05).

**Figure 6:**
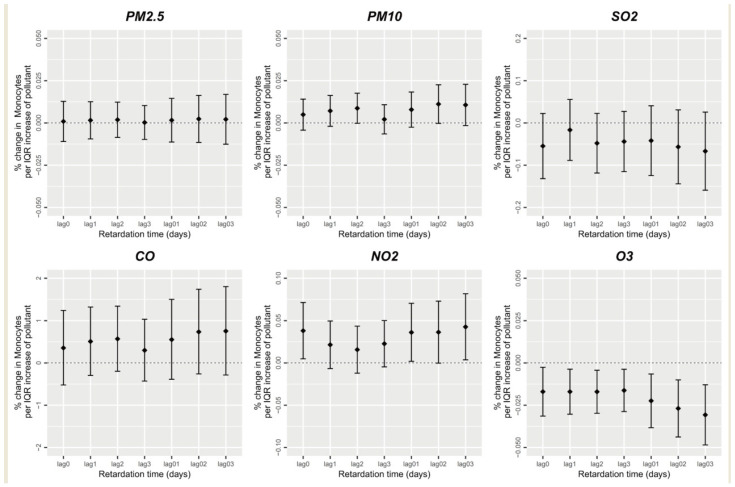
Short-term exposure to air pollutants and monocyte count.

Figure 6 depicts the association between short-term air pollution exposure and monocyte counts. Short-term NO_2_ exposure was significantly positively correlated with the participants’ peripheral blood monocyte numbers (*p* < 0.05), where every one-unit increase in the 0–3-day-lag NO_2_ average concentration led to a 0.043% increase in the peripheral blood monocyte number. However, short-term O_3_ exposure and the monocyte count were significantly negatively correlated (*p* < 0.05), which also reflected the fact that the average lag effect of short-term O_3_ exposure was generally larger than the single-day lag effect, and the average lag effect of 0–3-day-lag exposure was the strongest. The monocyte count decreased by 0.031% (*p* < 0.05) when the average concentration of 0–3-day-lag O_3_ increased by one unit. Nevertheless, PM_2.5_, PM_10_, SO_2_, and CO were not significantly correlated with peripheral blood monocyte numbers (*p* > 0.05).

## 4. Discussion

We analyzed the correlation between peripheral leukocyte distribution and air pollutant concentrations in 11,035 men from Beijing to reveal the effect of short-term air pollution exposure on peripheral leukocyte distribution in adult males. After adjusting for potential confounding factors (age, temperature, humidity, wind speed, air pressure, and season (heating and non-heating)), we determined that PM_2.5_, PM_10_, SO_2_, CO, NO_2_, and O_3_ were significantly correlated with at least one peripheral blood leukocyte subtype in the study population. The results indicated that the effects of air pollution exposure on the participants’ peripheral blood WBC distribution could be cumulative over time. Furthermore, the results suggested that it is necessary to study the effects of long-term (1-week or even 1-month) air pollution exposure on peripheral blood leukocyte distribution in the adult male population.

Our results indicated that short-term air pollution exposure in men induced inflammatory responses in vivo, leading to altered peripheral blood leukocyte distribution. Nevertheless, our results were not wholly consistent with those of previous studies based on different populations and sample sizes. We believe that short-term air pollution exposure (0–3 days) in the men was not significantly correlated with the total number of leukocytes in the peripheral blood. However, Zuurbie et al., Xu Gao et al., Yatera et al., and Gondalia et al. [21,22,23,24] demonstrated that short-term exposure to atmospheric PM decreased circulating WBC counts, while Steenhof et al. [25] demonstrated that short-term exposure to atmospheric particulate pollutants increased circulating WBC counts. The possible reason for these inconsistent results is that the atmospheric particle pollutant exposure time observed in the above studies was typically between 1 week and 1 month, while the atmospheric air pollutant exposure time observed in our study was relatively short (0–3 days). In this duration, the effect of air pollutants on the peripheral blood leukocyte count and classification might have been mainly due to peripheral blood leukocyte redistribution, while the effect of air pollutants on the bone marrow hematopoietic system and in turn the regulation of leukocyte distribution in systemic circulation may play a secondary role [26].

We did not observe a correlation between the participants’ short-term PM_2.5_ exposure and the peripheral blood neutrophil count, which was consistent with the results of Xu Gao et al. [22]. Contrastingly, Zuurbie et al. [21] suggested that short-term PM_2.5_ exposure would decrease the peripheral blood neutrophil count, while Steenhof et al. [25] and Riediker et al. [27] observed a positive correlation between short-term PM_2.5_ exposure and peripheral blood neutrophil counts. The potential reason for this inconsistent result was that the air pollution exposure time in our study differed from that of the above studies. Additionally, different studies involve different study populations and confounding factors, which may be possible reasons for the inconsistent results of our study and the relevant literature.

Our results suggest that short-term air pollution exposure (0–3-day lag; PM_2.5_, SO_2_, CO, NO_2_, and O_3_) in men was not significantly correlated with peripheral blood neutrophil counts. Conversely, the 2-day-lag PM_10_ was significantly positively correlated with neutrophil count, which was consistent with the results of Steenhof et al. [25]. The possible reason for this is that PM_10_ activates the inflammatory system in vivo [28], leading to neutrophil release from the reserve pool and resulting in increased neutrophils in the systemic circulation [29]. Additionally, we observed a significant negative correlation between short-term exposure to most air pollutants and peripheral blood eosinophils and basophils, which might have been due to the inhibitory effect of air pollutant exposure on the proliferation and differentiation of bone-marrow-related cells [30]. Furthermore, the heavy metals and chemicals in PM can block juvenile granulocyte differentiation into eosinophils and basophils [31,32].

Similar to the results of Salvi et al. [33] and Ma et al. [34], our results demonstrated that short-term PM_2.5_ exposure led to increased peripheral blood lymphocyte counts in the study population. The above results indicated that short-term air pollution exposure could activate the specific immune system in vivo, which then participates in the inflammatory process. We also observed a significant positive correlation between NO_2_ and the peripheral blood monocyte count, which was similar to the results of Xu Gao et al. [22].

Most previous studies only assessed the effects of air particulate pollutants on peripheral blood WBC counts. In addition to observing PM, we adjusted the confounding factors of environmental factors, namely temperature, humidity, air pressure, wind speed, season, and age. Consequently, the effects of gaseous pollutants such as SO_2_, CO, NO_2_, and O_3_ on the participants’ peripheral blood WBC distribution were analyzed, and the results were more specific and accurate than those of previous studies.

Generally, short-term air pollutant exposure can increase peripheral blood neutrophils, lymphocytes, and monocytes and decrease eosinophils and basophils. The reasons for this may be related to the inflammatory response induced by air pollutants [35] and the effects of exposure to the toxic heavy metals and chemicals in air pollutants, specifically in atmospheric PM, on peripheral blood leukocyte redistribution and bone marrow hematopoiesis. The inflammatory response induced by air pollution can generate the circular pool and reserve pool of redistribution, stimulate immature bone marrow granulocyte differentiation to neutrophils, and mildly inhibit eosinophil and basophil differentiation. Simultaneously, the immune response coordinates lymphocyte and mononuclear cell activation of the immune system and circulatory system, respectively, to participate in and promote the inflammation process. Furthermore, our results revealed that air pollutants exerted a significant time lag effect on the participants’ peripheral WBC distribution. We generally believe that it takes about 1 month for peripheral leukocytes to enter the peripheral blood from the bone marrow. However, most of the objects of the study were adult male patients in the outpatient department of our hospital who were in need of sperm extraction and had no history of blood or bone marrow transplantation. In addition, leukocytes are also divided into peripheral cisterns and circulating cisterns. Our routine blood test measured the number of peripheral white blood cells in the circulating pool. When the number fluctuates, the white blood cells in the marginal cisterna can play a role in maintaining the stable number of circulating white blood cells, so the number of white blood cells can reach the physiological homeostasis. In addition, there are mature pools and storage pools, so the body’s peripheral blood white blood cell reserves are extremely large. Clinically, we can know that in patients with hematologic diseases or patients with bone marrow suppression after chemotherapy, the number of peripheral blood white blood cells can be significantly increased 1–3 days after the injection of an ascending white needle. To some extent, our choice of short-term (0–3 days) observation is valid. However, long-term air pollution exposure may exert a more important influence on the peripheral leukocyte distribution in our study population, which requires confirmation in a subsequent study.

We performed a more comprehensive examination of the relationship between short-term air pollution exposure and the peripheral leukocyte distribution in adult males, providing data support for studying the effects of air pollution on male reproductive health. The participants were young or middle-aged men from the Reproductive Medical Center and the Department of Andrology of our hospital, where their main purpose for visiting was fertility problems. Generally, the men had relatively good nutrition, lower frequencies of smoking and drinking, and lower proportions of other systemic diseases. Additionally, the data filtering process excluded individuals who might have clinical diseases such as heart, liver, kidney, and endocrine disease. Therefore, the above factors were less likely to interfere with the results. The effect indexes of our study were peripheral blood WBC count and classification, which are routine indexes for the clinical observation of inflammation. The current clinical peripheral blood routine examination (mainly leukocyte count and classification) uses fully automated blood analyzers, which are high-throughput, lower-cost, simple, rapid, and high-accuracy and can quickly and efficiently process large quantities for sample analysis. Therefore, such an examination is well suited for the epidemiological assessment and study of preliminary inflammation in large populations.

This study has several limitations. First, inflammation is a complicated process involving multiple systems, cells, and biomolecules. In this study, the only inflammatory markers selected were the peripheral blood leukocyte count and classification. Thus, further exploration of the effect of air pollution on other inflammatory markers, such as the acute-phase protein C-reactive protein (CRP), cytokines, and more comprehensive observation of the inflammatory response and alterations induced by air pollution exposure is necessary. Second, the air pollution data in our study were the average levels of air pollution parameters in Beijing. Nevertheless, the wide Beijing area might have led to varying degrees of air pollution in different parts of the city. Furthermore, individual exposure sampling was not available; therefore, it was not possible to accurately quantify each participant’s individual air pollution exposure level. Additionally, indoor and outdoor air pollution levels demonstrate specific gradients and equilibrium times, and we were unable to collect and accurately estimate the individual participants’ daily indoor and outdoor activity times and whether they had air purification devices at home and the purification effect. The above factors all might have influenced the results. Third, we determined that a variety of air pollutants (including PM_2.5_, PM_10_, SO_2_, NO_2_, O_3_, and CO) might affect peripheral leukocyte distribution in men, but whether these pollutants interact and their synergistic effects on peripheral leukocyte distribution are topics that require further study.

## 5. Conclusions

We confirmed via a GAM that short-term air pollution exposure significantly affected peripheral leukocyte distribution in an adult male population. After adjusting for the influence of age, temperature, humidity, wind speed, air pressure, season, and other potential confounding factors, we determined that PM_2.5_, PM_10_, SO_2_, CO, NO_2_, and O_3_ were significantly correlated with at least one leukocyte subtype. Short-term air pollutant exposure elevated the peripheral blood neutrophil, lymphocyte, and monocyte counts and decreased eosinophil and basophil numbers. Furthermore, the results indicated that the effects of air pollution on peripheral blood WBC distribution in the adult male population may exert a cumulative effect over time. These results suggested that it is necessary to explore the changes in peripheral blood leukocyte distribution and the inflammatory process in men with longer exposure times, such as weeks or even months.

## Figures and Tables

**Table 1:** Descriptive statistical analysis result of meteorological factors and air pollutants in Beijing and the distribution of peripheral blood leukocytes in the study population.

	Mean	P_0.05_	P_0.25_	P_0.50_	P_0.75_	P_0.95_
Age	30.38	24	27	30	33	39
The distribution of peripheral blood leukocytes						
Leukocyte count	(×10^9^/L)					
WBC	6.676	4.620	5.650	6.550	7.600	9.173
Neutrophils	3.94	2.35	3.12	3.80	4.62	5.99
Eosinophils	0.0336	0.01	0.02	0.03	0.04	0.07
Basophils	0.1298	0.02	0.05	0.10	0.16	0.35
Lymphocytes	2.211	1.33	1.79	2.16	2.57	3.26
Monocytes	0.3618	0.22	0.29	0.35	0.42	0.55
Meteorology parameters						
Temperature (°C)	13.95	−3.30	2.80	15.30	24.30	28.86
Humidity (%)	49.88	19	33	50	66	82
Wind (m/s)	2.032	0.90	1.40	1.90	2.50	3.72
Pressure (hPa)	1012	997	1003	1012	1020	1029
Air pollutant index						
PM_2.5_ (μg/m^3^)	59.9	8	23	44	77	170
PM_10_ (μg/m^3^)	84.42	20	41	69	108	200.6
SO_2_ (μg/m^3^)	8.064	2	3	4	9	26
NO_2_ (μg/m^3^)	43.68	17	28	39	54	86
O_3_ (μg/m^3^)	96.86	12	52	82	138	220
CO (mg/m^3^)	0.99	0.3	0.5	0.8	1.1	2.5

## Data Availability

Weather data are available from: http://data.cma.cn/. The data of peripheral leukocyte distribution were obtained from the Peking University Third Hospital through the Health Information System, which has not deposited in publicly available repositories. Therefore, it is available from the corresponding author on reasonable request. Air quality data are obtained form: http://www.cnemc.cn/.

## Supplementary Materials

The following supporting information can be downloaded at: https://www.mdpi.com/article/10.3390/ijerph20064695/s1.

## Abbreviations

WBC, white blood cells; PM_10_, particulate matter ≤ 10 μm; PM_2.5_, particulate matter ≤ 2.5 μm; NO_2_, nitrogen dioxide; SO_2_, sulfur dioxide; CO, carbon monoxide; O_3_, ozone; CI, confidence interval; GAM, generalized additive models.

## References

[1] Chen B., Kan H. (2008). Air pollution and population health: A global challenge. Environ. Health Prev. Med..

[2] GBD RFC (2018). Global, regional, and national comparative risk assessment of 84 behavioural, environmental and occupational, and metabolic risks or clusters of risks for 195 countries and territories, 1990–2017: A systematic analysis for the Global Burden of Disease Study 2017. Lancet.

[3] Cohen A.J., Brauer M., Burnett R., Anderson H.R., Frostad J., Estep K., Balakrishnan K., Brunekreef B., Dandona L., Dandona R. (2017). Estimates and 25-year trends of the global burden of disease attributable to ambient air pollution: An analysis of data from the Global Burden of Diseases Study 2015. Lancet.

[4] Burnett R., Chen H., Szyszkowicz M., Fann N., Hubbell B., Pope C.A., Apte J.S., Brauer M., Cohen A., Weichenthal S. (2018). Global estimates of mortality associated with long-term exposure to outdoor fine particulate matter. Proc. Natl. Acad. Sci. USA.

[5] Shams S.R., Jahani A., Kalantary S., Moeinaddini M., Khorasani N. (2021). Artificial intelligence accuracy assessment in NO(2) concen-tration forecasting of metropolises air. Sci. Rep..

[6] Zhang Y., Zhang L., Wei J., Liu L., Wang Y., Liu J., Zhou P., Wang L., Ding Z., Zhang Y. (2021). Size-specific particulate air pollution and hospitalization for cardiovascular diseases: A case-crossover study in Shenzhen, China. Atmos. Environ..

[7] Park J., Kim H.-J., Lee C.-H., Lee H.W. (2021). Impact of long-term exposure to ambient air pollution on the incidence of chronic obstructive pulmonary disease: A systematic review and meta-analysis. Environ. Res..

[8] Ye J.-J., Wang S.-S., Fang Y., Zhang X.-J., Hu C.-Y. (2021). Ambient air pollution exposure and risk of chronic kidney disease: A systematic review of the literature and meta-analysis. Environ. Res..

[9] Vignal C., Guilloteau E., Gower-Rousseau C., Body-Malapel M. (2020). Review article: Epidemiological and animal evidence for the role of air pollution in intestinal diseases. Sci. Total Environ..

[10] Kim H., Kim W.-H., Kim Y.-Y., Park H.-Y. (2020). Air Pollution and Central Nervous System Disease: A Review of the Impact of Fine Particulate Matter on Neurological Disorders. Front. Public Health.

[11] Carré J., Gatimel N., Moreau J., Parinaud J., Léandri R. (2017). Does air pollution play a role in infertility?: A systematic review. Environ. Health.

[12] Krzastek S.C., Farhi J., Gray M., Smith R.P. (2020). Impact of environmental toxin exposure on male fertility potential. Transl. Androl. Urol..

[13] Cannarella R., Liuzzo C., Mongioì L.M., Condorelli R.A., La Vignera S., Bellanca S., Calogero A.E. (2019). Decreased total sperm counts in habitants of highly polluted areas of Eastern Sicily, Italy. Environ. Sci. Pollut. Res..

[14] Brook R.D., Rajagopalan S., Pope C.A., Brook J.R., Bhatnagar A., Diez-Roux A.V., Holguin F., Hong Y., Luepker R.V., Mittleman M.A. (2010). Particulate Matter Air Pollution and Car-diovascular Disease. Circulation.

[15] Halonen J.I., Zanobetti A., Sparrow D., Vokonas P.S., Schwartz J. (2010). Associations between outdoor temperature and markers of inflammation: A cohort study. Environ. Health.

[16] Altuwayjiri A., Taghvaee S., Mousavi A., Sowlat M.H., Hassanvand M.S., Kashani H., Faridi S., Yunesian M., Naddafi K., Sioutas C. (2021). Association of systemic inflammation and coagulation biomarkers with source-specific PM2.5 mass concentrations among young and elderly subjects in central Tehran. J. Air Waste Manag..

[17] El Haddad C., Gerbaka N., Hallit S., Tabet C. (2021). Association between exposure to ambient air pollution and occurrence of in-flammatory acne in the adult population. BMC Public Health.

[18] Elbarbary M., Oganesyan A., Honda T., Morgan G., Guo Y., Guo Y., Negin J. (2021). Systemic Inflammation (C-Reactive Protein) in Older Chinese Adults Is Associated with Long-Term Exposure to Ambient Air Pollution. Int. J. Environ. Res. Public Health.

[19] Eeden S.F.V., Hogg J.C. (2002). Systemic Inflammatory Response Induced By Particulate Matter Air Pollution: The Importance Of Bone-Marrow Stimulation. J. Toxicol. Environ. Health Part A.

[20] Wood S.N. (2003). Thin plate regression splines. J. R. Stat. Soc. Ser. B (Stat. Methodol.).

[21] Zuurbier M., Hoek G., Oldenwening M., Meliefste K., Krop E., Hazel P.V.D., Brunekreef B. (2011). In-Traffic Air Pollution Exposure and CC16, Blood Coagulation, and Inflammation Markers in Healthy Adults. Environ. Health Perspect..

[22] Gao X., Colicino E., Shen J., Kioumourtzoglou M.-A., Just A.C., Nwanaji-Enwerem J.C., Coull B., Lin X., Vokonas P., Zheng Y. (2019). Impacts of air pollution, temperature, and relative humidity on leukocyte distribution: An epigenetic perspective. Environ. Int..

[23] Yatera K., Hsieh J., Hogg J.C., Tranfield E.M., Suzuki H., Shih C.-H., Behzad A.R., Vincent R., Van Eeden S.F. (2008). Particulate matter air pollution exposure promotes recruitment of monocytes into atherosclerotic plaques. Am. J. Physiol. Circ. Physiol..

[24] Gondalia R., Holliday K.M., Baldassari A., Justice A.E., Stewart J.D., Liao D., Yanosky J.D., Engel S.M., Jordahl K.M., Bhatti P. (2020). Leukocyte Traits and Exposure to Ambient Par-ticulate Matter Air Pollution in the Women’s Health Initiative and Atherosclerosis Risk in Communities Study. Environ. Health Perspect..

[25] Steenhof M., Janssen N.A.H., Strak M., Hoek G., Gosens I., Mudway I.S., Kelly F.J., Harrison R.M., Pieters R.H.H., Cassee F.R. (2014). Air pollution exposure affects circulating white blood cell counts in healthy subjects: The role of particle composition, oxidative potential and gaseous pollutants–the RAPTES project. Inhal. Toxicol..

[26] Josefsson E., Tarkowski A., Caristen H. (1992). Anti-inflammatory properties of estrogen: I. In vivo suppression of leukocyte production in bone marrow and redistribution of peripheral blood neutrophils. Cell Immunol..

[27] Riediker M., Cascio W.E., Griggs T.R., Herbst M.C., Bromberg P.A., Neas L., Williams R.W., Devlin R.B. (2004). Particulate Matter Exposure in Cars Is Associated with Cardiovascular Effects in Healthy Young Men. Am. J. Respir. Crit. Care Med..

[28] Contiero P., Boffi R., Tagliabue G., Scaburri A., Tittarelli A., Bertoldi M., Borgini A., Favia I., Ruprecht A.A., Maiorino A. (2019). A Case-Crossover Study to Investigate the Effects of Atmospheric Particulate Matter Concentrations, Season, and Air Temperature on Accident and Emergency Presentations for Cardiovascular Events in Northern Italy. Int. J. Environ. Res. Public Health.

[29] Dorschner R.A., Lee J., Cohen O., Costantini T., Baird A., Eliceiri B.P. (2020). ECRG4 regulates neutrophil recruitment and CD44 expression during the inflammatory response to injury. Sci. Adv..

[30] Tsamou M., Vrijens K., Madhloum N., Lefebvre W., Vanpoucke C., Nawrot T.S. (2018). Air pollution-induced placental epigenetic al-terations in early life: A candidate miRNA approach. Epigenetics.

[31] Balali-Mood M., Naseri K., Tahergorabi Z., Khazdair M.R., Sadeghi M. (2021). Toxic Mechanisms of Five Heavy Metals: Mercury, Lead, Chromium, Cadmium, and Arsenic. Front. Pharmacol..

[32] Nguyen V.-T., Lee J., Qian Z.-J., Li Y.-X., Kim K.-N., Heo S.-J., Jeon Y.-J., Park W., Choi I.-W., Je J.-Y. (2013). Gliotoxin Isolated from Marine Fungus Aspergillus sp. Induces Apoptosis of Human Cervical Cancer and Chondrosarcoma Cells. Mar. Drugs.

[33] Salvi S., Blomberg A., Rudell B., Kelly F., Sandström T., Holgate S.T., Frew A. (1999). Acute Inflammatory Responses in the Airways and Peripheral Blood After Short-Term Exposure to Diesel Exhaust in Healthy Human Volunteers. Am. J. Respir. Crit. Care Med..

[34] Ma Q., Huang D., Zhang H., Wang S., Chen X. (2017). Exposure to particulate matter 2.5 (PM2.5) induced macrophage-dependent inflammation, characterized by increased Th1/Th17 cytokine secretion and cytotoxicity. Int. Immunopharmacol..

[35] Casey J.A., Wilcox H.C., Hirsch A.G., Pollak J., Schwartz B.S. (2018). Associations of unconventional natural gas development with de-pression symptoms and disordered sleep in Pennsylvania. Sci. Rep..

