# Supplementary material for: Associations between Short-Term Air Pollution Exposure and the Peripheral Leukocyte Distribution in the Adult Male Population in Beijing, China
Source: Int J Environ Res Public Health. 2023 Mar 7;20(6):4695. doi: 10.3390/ijerph20064695 (PMC10048523; doi:10.3390/ijerph20064695)

Figure S1. Time series analysis of the average daily concentration of air pollutants

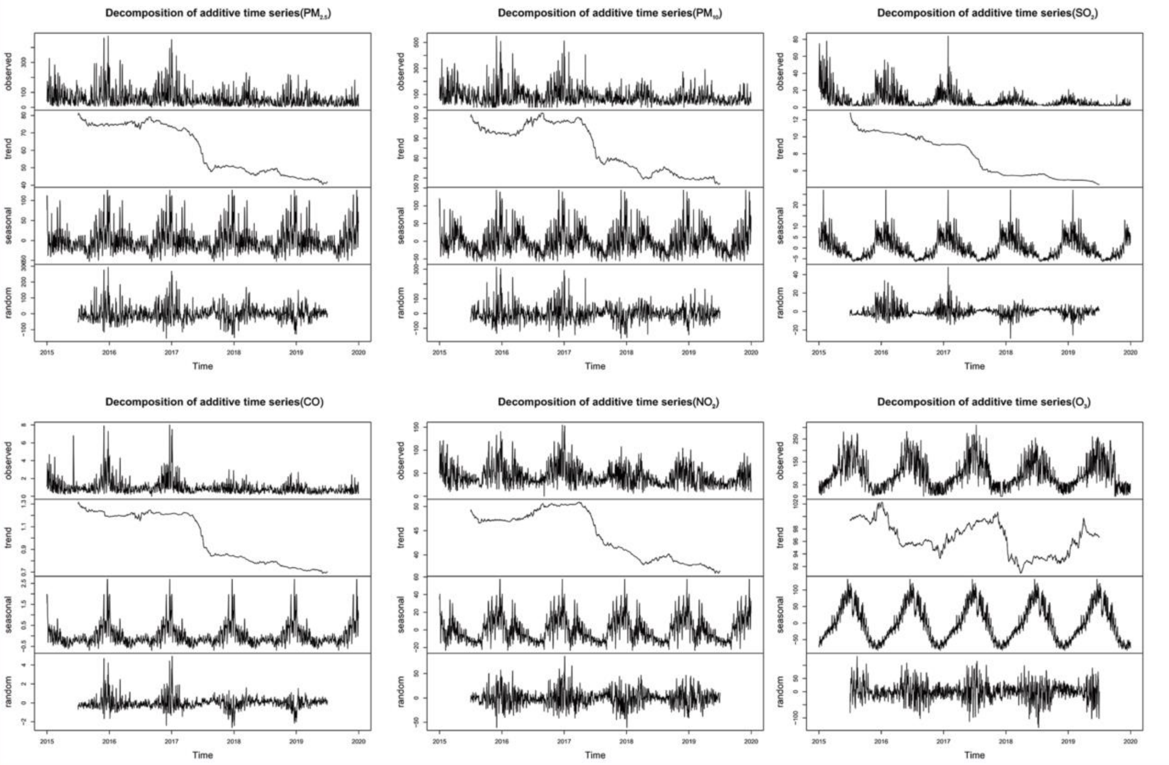

Figure S2. Spearman correlation analysis between the meteorological index and air pollutants

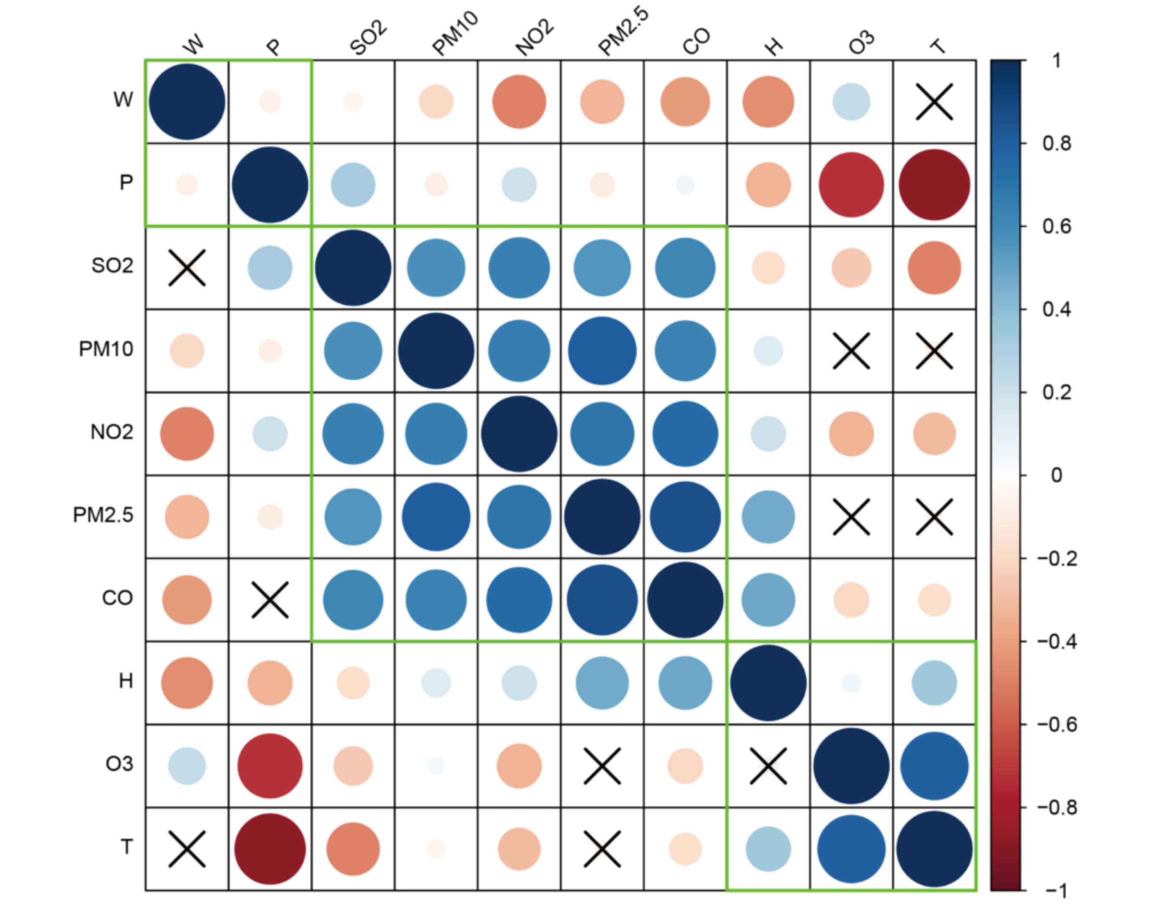

Supplement: Supplementary file 1 [file ijerph-20-04695-s001.zip › ijerph-2151095-supplementary.pdf]
